# Hydrodemethoxylation/Dealkylation on Bifunctional Nanosized Zeolite Beta

**DOI:** 10.3390/molecules26247694

**Published:** 2021-12-20

**Authors:** Margarita Popova, Ágnes Szegedi, Manuela Oykova, Hristina Lazarova, Neli Koseva, Magdolna R. Mihályi, Daniela Karashanova, Yavor Mitrev, Pavletta Shestakova

**Affiliations:** 1 Institute of Organic Chemistry with Centre of Phytochemistry, Bulgarian Academy of Sciences, Acad. G. Bonchev Str., bl. 9, 1113 Sofia, Bulgaria; Manuela.Oykova@orgchm.bas.bg (M.O.); Hristina.Lazarova@orgchm.bas.bg (H.L.); Yavor.Mitrev@orgchm.bas.bg (Y.M.); Pavletta.Shestakova@orgchm.bas.bg (P.S.); 2 Research Centre for Natural Sciences, Institute of Materials and Environmental Chemistry, Magyar Tudosok krt. 2, 1117 Budapest, Hungary; szegedi.agnes@ttk.hu (Á.S.); mihalyi.magdolna@ttk.hu (M.R.M.); 3 Institute of Polymers, Bulgarian Academy of Sciences, Acad. G. Bonchev Str., bl. 103A, 1113 Sofia, Bulgaria; koseva@polymer.bas.bg; 4 Institute of Optical Materials and Technologies, Bulgarian Academy of Sciences, Acad. G. Bonchev Str., bl. 109, 1113 Sofia, Bulgaria; dkarashanova@yahoo.com

**Keywords:** nanosized Beta zeolite, Ni/Ru/Pt catalysts, phenol synthesis, solid state NMR spectroscopy

## Abstract

Mono-, and bimetallic Ni-, Ru-, and Pt-modified nanosized Beta zeolite catalysts were prepared by the post synthesis method and characterized by powder X-ray diffraction (XRD), nitrogen physisorption, HRTEM microscopy, temperature-programmed reduction (TPR-TGA), ATR FT-IR spectroscopy, and by solid-state MAS-NMR spectroscopy. The presence of nanosized nickel-oxide, ruthenium-oxide, and platinum species was detected on the catalysts. The presence of Brønsted and Lewis acid sites, and incorporation of nickel ions into zeolite lattice was proven by FT-IR of adsorbed pyridine. The structural changes in the catalyst matrix were investigated by solid state NMR spectroscopy. The catalysts were used in a gas-phase hydrodemethoxylation and dealkylation of 2-methoxy-4-propylphenol as a lignin derivative molecule for phenol synthesis.

## 1. Introduction

Due to the gradual increase in energy demand and the depletion of fossil carbon resources, the conversion of lignocellulosic biomass to fuels and chemicals has recently become the focus of research [1,2,3,4,5]. Lignocellulose is mainly composed of polymers of carbohydrates (cellulose and hemicellulose) and aromatics (lignin). Significant progress has been made in the production of value-added compounds from platform molecules obtained by acidic hydrolysis of cellulose and hemicellulose. However, valorization of lignin remains a major challenge due to the structural complexity and recalcitrance of the oxygenated p-propylphenol polymer [6]. The main lignin sources are the paper and pulp industry, as well as biorefineries producing bioethanol from cellulose. Despite of its emerging applications, such as emulsifier, binding agent, rheology modifier, carbon fiber precursor, etc., only 2% of lignin is commercialized. The rest is burned to produce cheap energy [7]. The depolymerization of lignin is the first step of its valorization, which leads to the formation of a complex mixture of phenolic compounds. The catalytic processes of selective demethoxylation and dealkylation leads to transformation of substituted phenols to phenol. Efficient dealkylation/demethoxylation of alkylphenols derived from lignocellulose was reported on bimetallic catalysts with metal active sites and Lewis and/or Brønsted sites (Pt/γ-Al_2_O_3_, Pt-, Ru- zeolite ZSM-5, and ZSM-22, beta) [8,9,10,11,12,13,14,15,16]. The most efficient catalyst could selectively catalyze demethoxylation and de-alkylation processes and the mixture of substituted phenols will be converted into phenol. The phenol production is one of the most important industrial processes reaching an annual production of 8.9 million tons [17].

The application of nanosized bifunctional catalysts can improve the conversion of alkyl phenols and the selectivity to phenol production. Selection of metal species is of great importance for the demethoxylation and dealkylation processes. Noble metals, such as Ru, Rh, Pd, and Pt are highly active for the dealkylation processes. Alternatively, less-costly metals such as Ni, Co, and Fe have been studied for bio-oil HDO. There are data presenting USY zeolite as a support, and they have demonstrated the benefit of USY supported transition metals on more than one bio-oil model compound. However, the micropores of the zeolite hinder the reactant access. The application of metals supported mesoporous acid zeolite catalysts could be a promising alternative to the conventional ones.

In this study, a nanosized zeolite Beta was modified with metals (Ni/Ru/Pt) for the preparation of monometallic and bimetallic zeolite catalysts. The obtained catalysts were studied in the hydrodemethoxylation and dealkylation of 2-methoxy-4-propylphenol.

## 2. Results and Discussion

### 2.1. Physico-Chemical Properties

The pristine nanosized beta zeolite has peculiar textural properties. It is composed of 150–500 nm sized spherical particles, which are not single zeolite crystals, but agglomerated of 10–20 nm nanoparticles. Among the agglomerates there are mesopores, providing some hierarchical structure to the zeolite.

**Figure 1:**
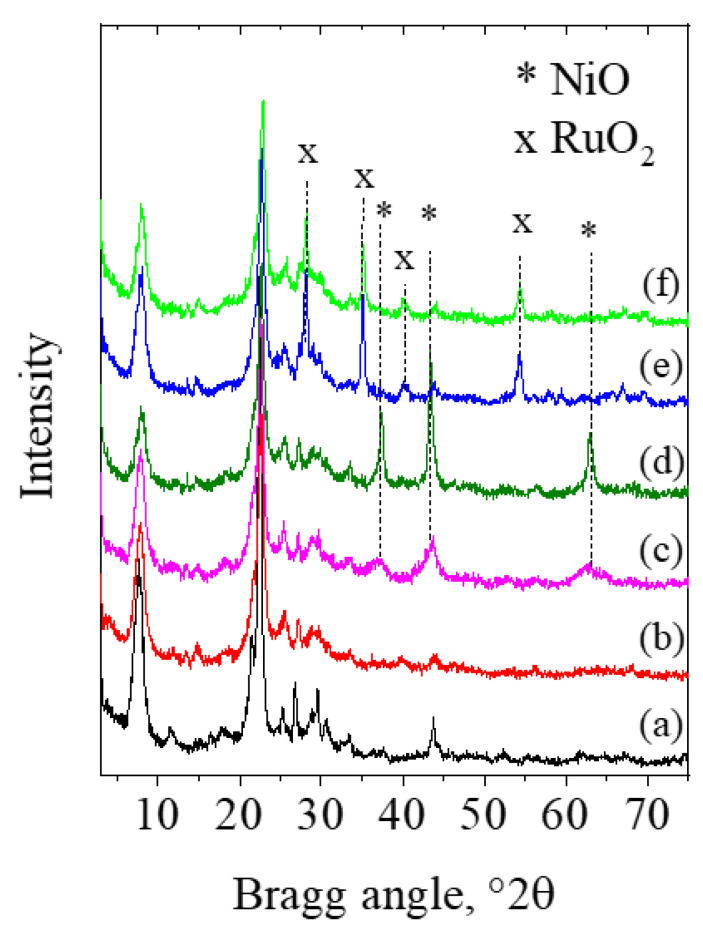
X-ray powder diffraction patterns of the studied catalysts: (**a**) initial zeolite Beta, (**b**) 1Pt/HB, (**c**) 10Ni/HB, (**d**) 10Ni1Pt/HB, (**e**) 5Ru/HB, (**f**) 5Ru1Pt/HB.

The crystalline phases of the zeolite Beta catalysts were identified by X-ray powder diffraction, and the results are shown in Figure 1. According to the patterns crystalline structure of the zeolite was preserved, and reflections characteristic for RuO_2_ and NiO nanoparticles on the external surface or among the voids of nanosized zeolite structure were found in the corresponding catalysts. Reflections of metallic platinum cannot be detected in Pt-containing preparations. It indicates that Pt particles that formed are quite small, below 5 nm. Incorporation of ionic Pt species into the lattice ion-exchange positions also cannot be excluded [18]. The crystallite size of metal oxide particles was calculated by the Scherrer equation applying profile fitting method. It was found that NiO particles are with the size of ~5 nm for 10Ni/HB and 26 nm for 10Ni1Pt/HB (Table 1). The formation of ionic Pt species hinders the strong nickel interaction with the support leading to the formation of bigger NiO particles on 10Ni1Pt/HB. Similar or somewhat smaller RuO_2_ particles (26 nm) are formed on the 5Ru/HB sample then on the bimetallic 5Ru1Pt/HB (29 nm) one. This effect could be explained by the preparation procedure of bimetallic catalysts in which the modification with Pt is the first step followed by modification with Ni and Ru for 10Ni1Pt/HB and 5Ru1Pt/HB, respectively. The succession of the modification procedure could benefit stronger interaction with Pt precursor with the unmodified surface of the initial Beta zeolite. The calculated size of the metal oxide particles is higher than the pore size of the support, which indicates that most of them are on the external surface of the zeolite. The high surface area and the nanosized morphology of zeolite particles predetermine the high dispersion of metal oxide nanoparticles on the modified catalysts.

**Figure 2:**
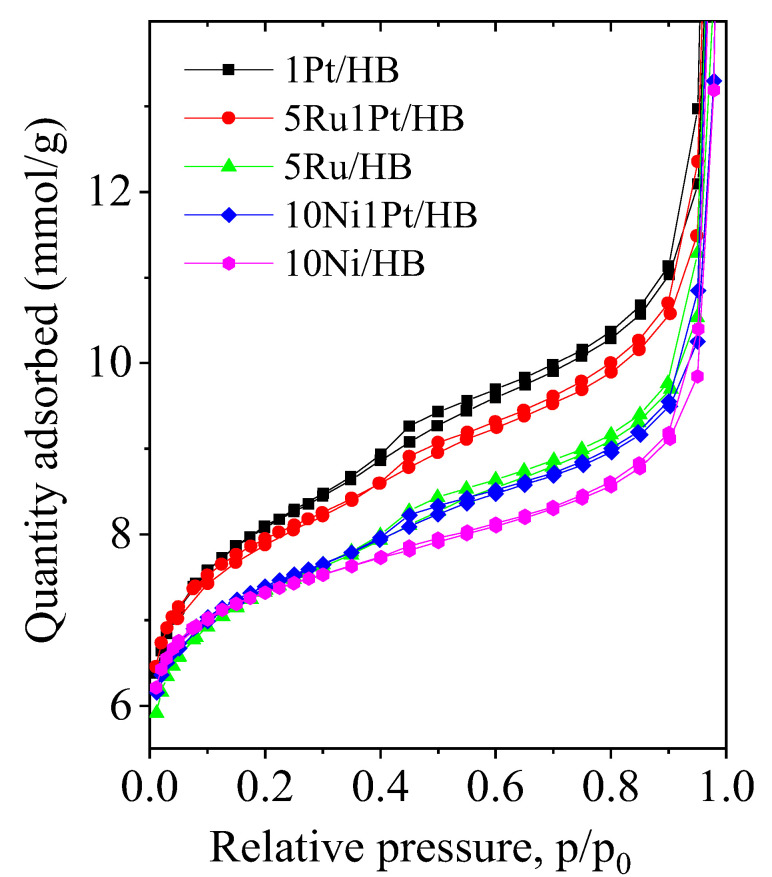
Nitrogen adsorption/desorption isotherms of Ni-, Ru-, and Pt-modified HB zeolites.

The nitrogen adsorption/desorption isotherms of the initial Beta zeolite and its metal-modified varieties are shown in Figure 2. The calculated textural parameters are in Table 1. The isotherms are of type IV, typical for hierarchical zeolites, with some small hysteresis loop between p/p_0_ = 0.4–0.6, characteristic for mesopores, probably in connection with the defected structure of the zeolite. By impregnation with noble and transition metals, somewhat decreased specific surface area and total pore volume can be observed (Table 1). It indicates that impregnating salts are penetrated the silica channels and the formed metal oxide nanoparticles are partly clogged them; occupation of ionic positions in zeolite lattice by metal cations is also a possible explanation.

**Figure 3:**
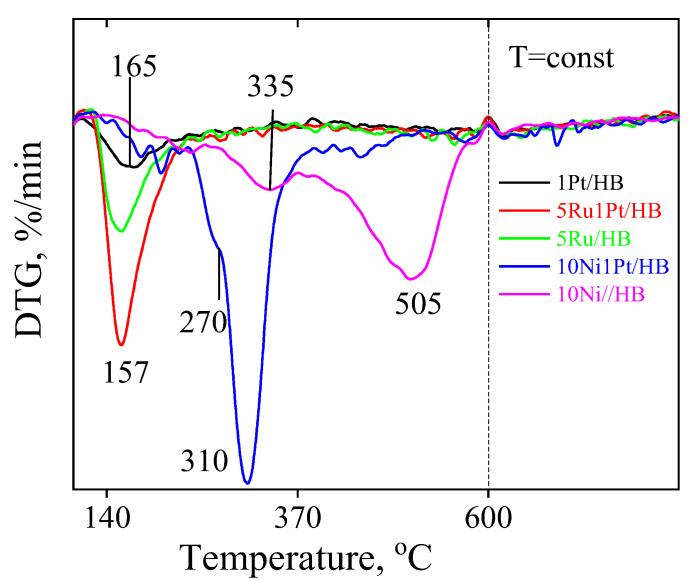
TPR profiles of the Ni-, Ru-, and Pt-modified nanosized Beta zeolites.

TPR-TG curves of the metal-containing mono- and bimetallic catalysts are displayed in Figure 3. TPR signal of 5Ru1Pt/HB sample shows the presence of one reduction peak below 200 °C in likewise the results of Gamliel et al. [19], with different intensities indicating the distinct reducibility of the metal oxide particles, 100% for 5Ru1Pt/HB. The calculated extent of reduction in RuO_2_ particles in 5Ru/HB is 63%. However, our TEM results detected some metallic Ru particles. In order to calculate the real reducibility, we performed a consecutive temperature-programmed reduction 1 oxidation-reduction 2 (TPR1-TPO-TPR2) experiment. The calculated extent of reduction in the second TPR2 experiment corresponds to 96% (Table 1). We assume that the strong interaction of Pt with the support stabilizes the dispersion of RuO_2_ particles. In the TPR curves of nickel-containing samples intensive peaks centered at higher temperatures, between 335 and 505 °C for 10Ni/HB and a much lower one between 270 and 310 °C for 10Ni1Pt/HB, can be distinguished. The lower temperature step most likely can be identified as a reduction in NiO particles, finely dispersed on the external surface of zeolite. Whereas the peak at elevated temperatures is associated with the more difficult reduction in ionic species situated in the zeolite lattice. The latter ones can be formed by ion-exchange during the wet impregnation procedure. In contrast to 10Ni1Pt/HB sample the reduction in 10Ni/HB is incomplete up to 600 °C (90%), which fact also supports the formation of hardly reducible ionic Ni species in the zeolite lattice. It seems that the presence of platinum has advantageous effect on reducibility of RuO_2_ and NiO on zeolite Beta: the reducibility of Ru was increased, whereas nickel could be reduced at much lower temperature. Platinum is also reduced below 200 °C in this system, which suggest that Pt is in the form of easily reducible oxidic nanoparticles [20]. Pt in ion-exchange positions would be reducible over 250 °C.

The band at 3735 cm^−1^ in the ATR FTIR spectrum [21] of the parent Beta zeolite is ascribed to the presence of internal SiOH groups (Figure S1). The shift of this band is registered for the modified zeolites and it is due to the strong interaction of the applied metals with the silanol groups of the support.

**Figure 4:**
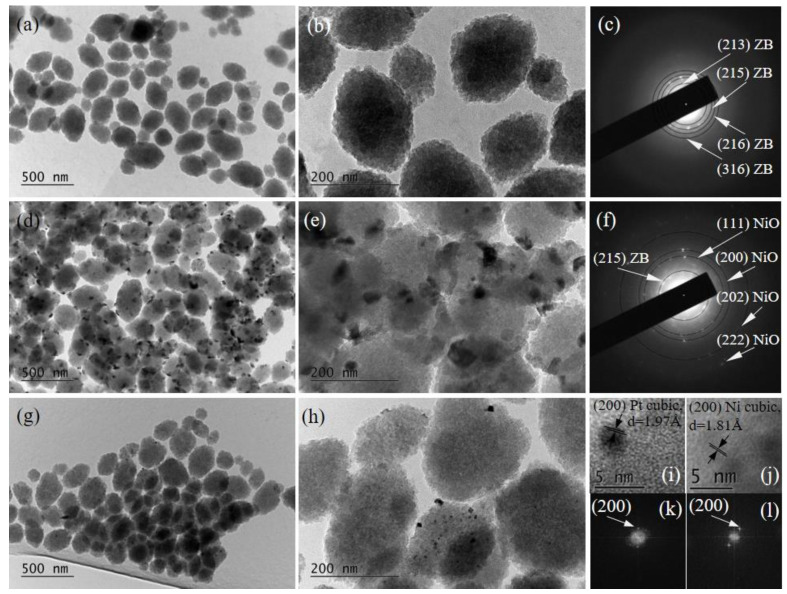
Bright Field TEM (BFTEM) images of zeolite Beta (**a**,**b**), 10Ni1Pt/HB (**d**,**e**) and 5Ru1Pt/HB (**g**,**h**). SAED patterns of zeolite Beta (**c**) and 10Ni1Pt/HB (**f**). HRTEM images and the corresponding Fast Fourier Transform (FFT) patterns of 10Ni1Pt/HB (**j**,**l**) and 5Ru1Pt/HB (**i**,**k**) samples.

TEM micrographs and SAED patterns, as well as HRTEM images and the belonging FFT patterns of the initial and modified Beta zeolites are presented in Figure 4. A large-scale view at magnification of 10,000× of the aggregated particles is shown in Figure 4a,d,g, whereas images made at higher magnification (Figure 4b,e,h) demonstrate in more detail the shape and size of particles and the way of metal nanoparticle deposition on the zeolite surface. The zeolite particles are of elliptic shape and the sizes are between 100 and 200 nm for the longer diameter (Figure 4b). The metallic nanoparticles are rather spherical, and their size is below 10 nm, as it is presented for Pt NPs in Ru-Pt sample (Figure 4i). These nanoparticles tend to coalesce, thus forming aggregates with irregular shapes, more expressed in the Ni-Pt sample (Figure 4e).

SAED analysis prove the presence of tetragonal beta zeolite (# 91, database of the Zeolite Association), (Figure 4c) and cubic NiO (# 96-101-0094, Crystallography Open Database, COD), (Figure 4f). These data are in accordance with the XRD results, but in addition HRTEM reveals the presence of cubic metallic Ni (# 96-901-3035, COD), (Figure 4j,l) as well as cubic Pt (# 96-101-1104, COD), (Figure 4i,k) phases in Ni-Pt and Ru-Pt samples, respectively. The metal nanoparticles deposition on the external surface which was assumed from XRD data is supported also by TEM measurements.

Additional information received by TEM analysis of the monometallic Ru sample is provided in Figure S2 in Supplementary materials. The TEM micrographs presented in Figure S2a,b reveal the morphology of the particles in the 5Ru/HB sample. The zeolite particles with their ellipsoidal shape and weak electronic contrast, known from Figure 4a,b of the main paper, are well seen. The deposited on their surface, predominantly rod-like, well-faceted crystals with higher electronic contrast and sizes from 10 to 100 nm are also visualized. The HRTEM analysis proves that these well faceted particles represent RuO_2_ crystals, according the measured interplanar distance d = 2.24 Å between the lattice fringes in Figure S2e). This value coincides with the presented in Crystallography Open Database (COD), file # 96-100-0059 for (200) family of planes of tetragonal RuO_2_. At the same time, second type of particles is found in this sample. Spherical-like and less than 10 nm in diameter, they are not visible in the micrographs at magnifications up to 40,000× because of their small sizes. The measured in Figure S2f interplanar distance for one particle of this type d = 2.21Å coincides well with the interplanar distance of (111) family of planes of metallic Ru (COD # 96-153-4915). Additionally, ruthenium oxide is identified together with the zeolite phase in the sample Ru/HB also by indexing the SAED pattern in Figure S2c, and the FFT pattern in Figure S2d, while the metallic Ru is proved also by the FFT pattern in Figure S2g.

The parent, and the Ru, Pt, and Ni modified HB zeolites were investigated by ^29^Si and ^27^Al NMR spectroscopy. The five different structural environments of the tetrahedrally coordinated Si atoms, denoted as Si(nAl), where n = 0, 1, 2, 3, and 4 is the number of Al atoms sharing oxygen with the SiO_4_ unit, have characteristic chemical shifts in the ^29^Si NMR spectra. In addition, the relative areas of the signals for the different Si(nAl) species reflect the composition of the zeolite framework. The single pulse ^29^Si NMR spectra were quantitatively analyzed and the NMR signal areas of the different Si structural units were used to calculate the framework Si/Al ratio according to following equation [19]:

(1) $$Si/T=\frac{\sum_{n=0}^{4}I_{\mathrm{Si}}{}_{\left(nT\right)}}{\sum_{n}^{4}0.25{\mathrm{nI}}_{\mathrm{Si}(\mathrm{nT})}}$$

where I_t_ is the total area of the spectrum and I_Si__(nAl)_ is the contribution of Si atoms with nAl neighbors in the second coordination sphere of the SiO_4_ units.

**Figure 5:**
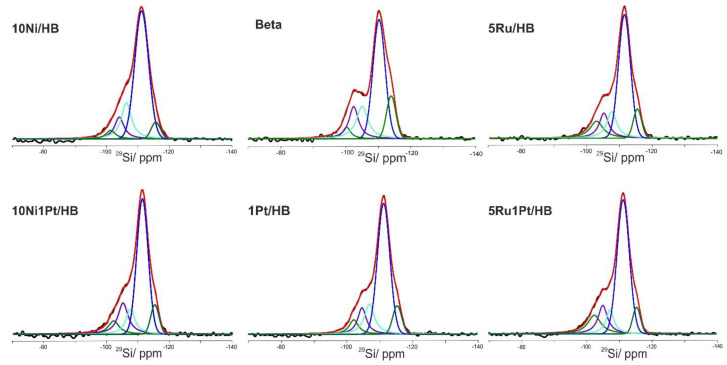
Experimental (black) and simulated (red) single-pulse ^29^Si NMR spectra of the studied zeolites. The individual contributions of the different Si environments are given with colored lines.

**Figure 6:**
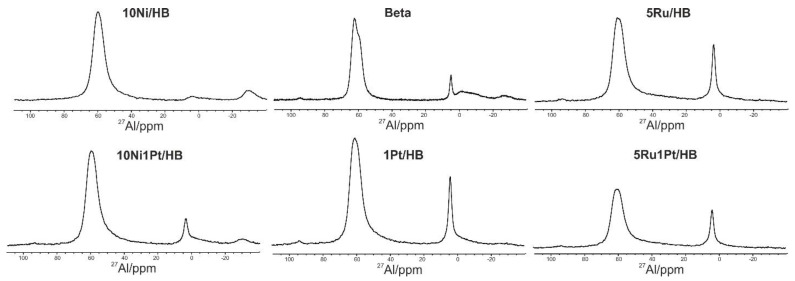
^27^Al NMR spectra of the studied zeolites.

The single pulse ^29^Si spectra and relative fractions of the different Si structures obtained by the deconvolution and fitting the spectra with the DMFit software are presented in Figure 5 and Table 2, respectively. The spectra of all materials showed five main resonances with chemical shifts centered at about −101 ppm, −105 ppm, −107 ppm, −110 ppm, and −114 ppm. The resonances at −110 ppm and −114 ppm are characteristic for Si(0Al) species originating from two crystallographic tetrahedral Si(0Al) sites. The signal at −107 ppm is typically assigned to Si(1Al) units from ((≡Si–O)_3_≡Al–(OH)–Si≡) structural motifs with bridged hydroxyl groups. We suggest that the resonance at −105 ppm could be associated with Si(1Al) species from defect framework sites where the Si atom is bound to tri-coordinated Al species. The resonance at −102 ppm indicates that some silanol groups Si(1OH) are also present as defect sites in the zeolite matrix [18,20,22]. For the calculation of the Si/Al ratio the total fraction of the Si(0Al) total was considered as sum of the contributions of the Si(0Al) resonances at −110 ppm and −114 ppm, and the resonances for the Si(1OH) at −102 ppm. The framework Si/Al ratio of 21 was determined for the parent H-Beta zeolite. This ratio is higher as compared to the total Si/Al ratio of 16 measured by ICP analysis, which could be explained by the fact that ICP measures both the FAl and EFAl, while ^29^Si NMR does not take into account the aluminum originating from EFAl species. We assume that during the loading of metal, part of the EFAl species is introduced into the zeolite framework, and therefore the process of “healing” of zeolite framework occurs, resulting in decrease in the Si/Al ratio. This suggestion is confirmed by the ^27^Al NMR data (Figure 6) indicating that for all metal impregnated samples except 5Ru/HB, the fraction of FAl species increases while the fraction of the EFAl species is decreased (Table 2, last two columns). For the 5Ru/HB sample, the Si/Al ratio is still lower as compared to the parent sample, indicating that the “healing” process occurs; however; the ratio of EFAl to FAl species remains the same as for the parent zeolite. This apparent contradiction could be explained with distortion of the symmetry of the environment of some Al sites upon introduction of Ru. Previous studies demonstrated that in low symmetry environment the resonances of Al nuclei can be very broad and difficult to detect due to second-order interactions between the electric field gradient at the nucleus and the nuclear quadrupole moment of ^27^Al, resulting in fast relaxation of the Al nuclei. Therefore, we suggest that part of the signal originating from these distorted FAl sites (NMR “invisible” Al sites) is not fully detected, which gives lower apparent fraction for the FAl spices. The presence of distorted Al species is confirmed in the ^27^Al spectrum by the observed broad low intensity hump in the region 35-50 ppm, partially overlapped with the main FAl resonance at around 60 ppm (Figure 6).

The ^29^Si NMR spectra of mono- and bimetallic zeolite systems show the presence of the same type of Si structural units as in the parent Beta material, however with different quantitative distribution (Table 2). As a general trend the total fraction of the Si(0Al) species (the resonances at −110 and 114 ppm) was similar to that in the parent Beta while the total amount of Si(1Al) species was increased. A decrease in the SiOH groups resonance at −101 ppm was also detected.

The presence of SiOH groups was identified by the ^1^H→^29^Si CP MAS spectroscopy, which is based on transfer of polarization from sensitive spins (^1^H) to nuclei with low sensitivity (^29^Si) via through space dipole–dipolar interactions. Figure S3 shows as an example the ^1^H→^29^Si CP MAS spectra of Beta and 10Ni/BH samples overlaid with their ^29^Si single pulse spectra for comparison. In the ^1^H→^29^Si CP spectra of both samples the most enhanced resonance is at −101 ppm, indicating the presence of silanol groups. The is also a slight enhancement of the Si(0Al) and the Si(1Al) signals, due to possible magnetization transfer from the neighboring silanol protons, bridged hydroxyl protons, and/or coordinated water molecules. The enhancement of the Si(0Al) resonances is more pronounced in the parent Beta material while it could be considered as negligible in the10Ni/BH sample. Although the ^1^H→^29^Si CP spectra cannot be interpreted quantitatively, this result confirms that the number of Si-OH groups in mono- and bimetallic zeolites is generally lower as compared to the parent material.

The coordination state of Al nuclei in the zeolite framework was investigated by ^27^Al NMR (Figure 6). The parent Beta zeolite shows two partially overlapped resonances at −60 ppm that are characteristic for the tetracoordinated framework Al sites (FAl) with different symmetry. The presence of extra-framework Al species EFAl species represented by a narrow resonance at ~4 ppm and a broad higher field signal centered at ~−5 ppm. The simultaneous presence of FAl and EFAl was observed also by Majano et al. [23] when the same type of zeolite Beta was heat treated for template removal. Formation of octahedrally coordinated aluminum in hydrated samples can be explained also by coordination of water and/or silanol groups to tricoordinated aluminum atoms. The latter species can act as Lewis acid sites when dehydrated, and can be ‘healed’, i.e., converted to tetrahedral ones by dehydration or by incorporation of ammonia [24] or other metal cations into the lattice, as evidenced by our former study with silver ions [25].

The modification of parent Beta zeolite with Ru, Ni, and Pt results in some structural changes depending on the applied metals. The ^27^Al spectra of all mono- and bimetallic zeolites display similar signals as parent HB, except Ni modified ones. Different degrees of structural changes were observed as result of modification of parent Beta zeolite with Ru, Ni, and Pt. The ^27^Al spectra of all mono- and bimetallic zeolites display similar signals as parent HB, except Ni modified ones. A broad resonance at around 40–38 ppm is mainly associated with additional structural transformations in the impregnated materials due to a distortion of the symmetry of the environment of some Al sites [23,24,25,26,27,28,29]. The fractions of FAl and EFAl species are given in the last two columns of Table 2. The parent HB catalyst has around 20% of EFAl sites, a huge part of it probably being hydrated tricoordinated aluminum, as evidenced by the significant intensity decrease in the 0 ppm signal by Ni impregnation (10Ni and 10Ni1Pt/HB samples). The lowest content of the EFAl species was registered for 10Ni/HB sample, which can be explained by the ion-exchange of nickel cations into the zeolite matrix during the impregnation process. Ni ions can be coordinated to trigonal aluminum and form tetrahedral sites. Ion-exchange with nickel ions was evidenced also by FT-IR spectroscopy of adsorbed pyridine (see below). The FAl/EFAl ratio in the other three samples 1Pt/HB, 5Ru/HB, and 5Ru1Pt/HB was similar to those observed in the parent Beta material, indicating no ion exchange with Ru or Pt.

**Figure 7:**
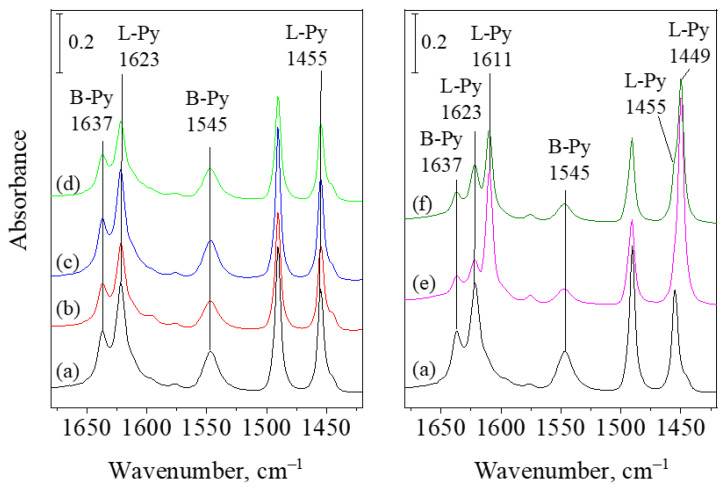
FT-IR spectra of adsorbed pyridine on the studied catalysts: (**a**) parent zeolite Beta, (**b**) 1Pt/HB (**c**) 5Ru/HB, (**d**) 5Ru1Pt/HB, (**e**) 10Ni/HB, (**f**) 10Ni1Pt/HB. Py (6 mbar) was adsorbed on 400 °C dehydrated samples at 200 °C and desorbed at 200 °C in high vacuum.

Acidity of the catalysts can be investigated by FT-IR spectroscopy by adsorption of a base, such as pyridine (Py). The latter method can help to explore the Brønsted and Lewis acidic character of the catalysts [30]. Figure 7 represents the FT-IR spectra of the studied samples following Py adsorption. Ring vibrations 8a, 8b, and 19b of Py give characteristic adsorption bands between 1700 and 1400 cm^−1^ [31]. Electron deficient surface Lewis acid sites (L-Py) interact with lone pairs of nitrogen in Py, or pyridinium ion is formed, when Brønsted acidic protons of zeolites (B-Py) reacts with Py. The band pair at 1545/1635 cm^−1^ is characteristic for B-Py sites, and L-Py bands can be found at 1445–55 and 1610–23 cm^−1^ values. Brønsted acidic sites are created in zeolites by balancing the negative framework charge of tetrahedrally coordinated Al(III) species with a proton, also known as bridged hydroxyl groups ((≡Si–O)_3_≡Al–(OH)–Si≡). Lewis acid bands are formed by interaction of Py with lattice cations like extra-framework AlO^+^ or other metal ionic species. By Py adsorption one can get information about the amount of acid sites and their strength can be characterized by the temperature of desorption. In highly acidic zeolites Py is retained even at 400 °C by Brønsted and Lewis sites.

**Figure 8:**
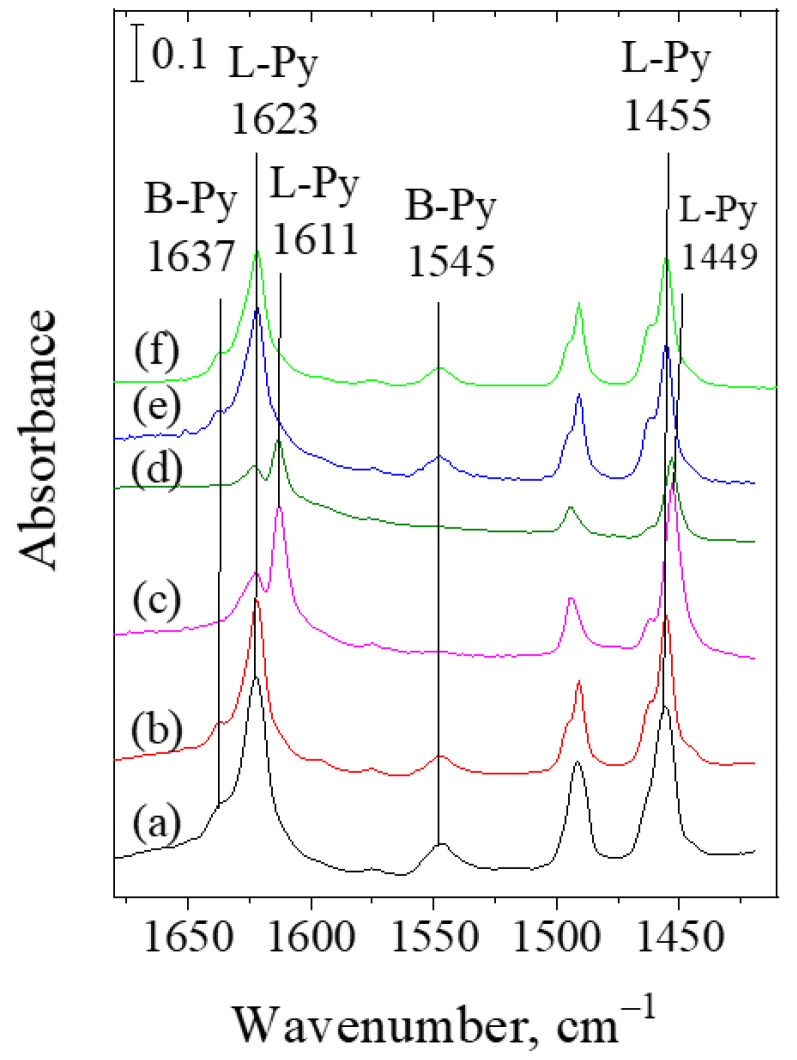
FT-IR spectra of adsorbed pyridine on the studied catalysts: (**a**) parent zeolite Beta, (**b**) 1Pt/HB, (**c**) 10Ni/HB, (**d**) 10Ni1Pt/HB, (**e**) 5Ru/HB, (**f**) 5Ru1Pt/HB. Py (6 mbar) was adsorbed on 400 °C dehydrated samples at 200 °C and desorbed at 400 °C in high vacuum.

As formerly evidenced by NMR results, both Lewis and Brønsted type acid sites are detectable on HB (Figure 8a), however, compared to other zeolites, the Lewis acid centers are rather characteristic for defective structure of Beta than for extra framework AlO^+^ species. Thus, the 1455/1623 cm^−1^ band pair can be associated with Py coordinatively bound to tri-coordinated aluminum species in the defect sites of the zeolite.

Impregnation with noble metals slightly decreased the Brønsted acidity of the catalysts (Figure 7b–d), most probably in association with some structure deterioration of the zeolite supported by XRD results. However, in the case of the nickel-containing samples (Figure 7e–f) decreased intensity of the L-Py bands and turn up of new, strong bands at 1449/1611 cm^−1^ proves some structural changes, namely, the presence of Ni^2+^ ions at cationic positions of the zeolite lattice. It seems that during the impregnation procedure ion exchange occurred. Nickel ions can replace the protons in bridged hydroxyl groups or can be connected to silanol groups neighboring the tri-coordinated aluminum species in the defect sites. The latter results in the ‘healing’ of the zeolite framework, i.e., changing of coordination of octahedral aluminum to tetrahedral, as supported also by ^27^Al NMR results (Figure 6). The decreased intensity of 1545 cm^−1^ band shows that only about the one third of the original bridging hydroxyls remained by incorporation of nickel ions into the zeolite lattice. When Ni is impregnated on Pt-containing zeolite, the incorporation of nickel into cationic positions is incomplete, proved by the less intense 1450/1611 cm^−1^ Lewis bands, and more intense 1455/1623 cm^−1^ ones (Figure 8e) compared to 10Ni/HB catalyst. This observation is in line with XRD results, detecting higher amount of NiO on the external surface of platinum containing zeolite Beta, and by ^27^Al NMR investigations shoving higher amount of octahedral aluminum species (Figure 6). Ni incorporation influenced not only the number of acid sites but also the strength of acidity, because Py was totally desorbed at 400 °C from the latter samples, in contrast to other studied catalysts (Figure 8c–d). Based on FT-IR spectroscopic method no evidence for entering of Pt or Ru ions into cationic positions could be found.

### 2.2. Catalytic Activity for Phenol Preparation

**Scheme 1:**
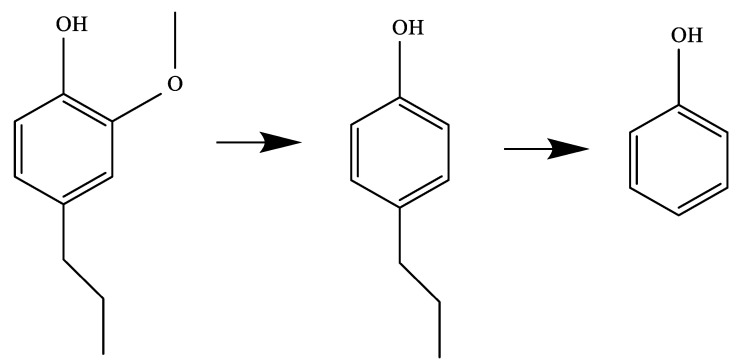
2-Methoxy-4-propylphenol transformations on acid and metallic sites.

**Figure 9:**
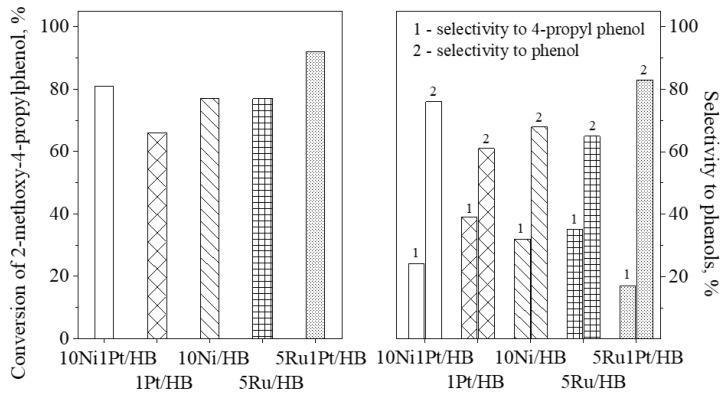
Catalytic activity of Ni-, Ru-, Pt-modified Beta zeolites in hydrodemethoxylation and dealkylation of 2-methoxy-4-propylphenol at 300 °C.

The prepared catalysts were investigated in hydrodemethoxylation and dealkylation of 2-methoxy-4-propylphenol. Demethoxylation of 2-methoxy-4-propylphenol leads to 4-propylphenol, followed by dealkylation to give phenol (Scheme 1). Liao et al. [32] showed that on zeolite beta in the presence of steam and in the absence of hydrogen and a metal component, not only dealkylation but isomerization, transalkylation and disproportionation of 4-propylphenol occur, especially at lower temperatures. The conversion of 2-methoxy-4-propylphenol and the selectivity to 4-propylphenol and phenol are compared at 300 °C for all catalysts (Figure 9). The isomerization, disproportionation and transalkylation of 4-propylphenol were negligible in the applied conditions.

2-methoxy-4-propylphenol conversion and phenol selectivity are higher on bimetallic catalysts than on monometallic catalysts (Figure 9A). Of the bimetallic catalysts, 5Ru1Pt/HB showed better catalytic performance. The presence of acidic and metallic centers is important for the transformations of 2-methoxy-4-propylphenol to phenol. Acidic and metallic centers of the catalysts play crucial role in transformation of 2-methoxy-4-propylphenol to phenol. Metal species selectively catalyze the hydrogenolysis of the methoxy group promoting the formation of 4-propylphenol. In addition, dealkylation of alkyl phenols to phenol proceeds on the acid sites (Scheme 1). RuO_2_ nanoparticles of 5Ru1Pt/HB catalyst showed higher reducibility, most probably owing to the interaction with platinum, resulting in the highest hydrogenolysis activity. It seems that the bimetallic Ru and Pt modification of zeolite HB led not only to higher activity but also to improved selectivity to phenol. This is explained by the peculiar properties of the zeolite-based catalysts, i.e., the combined effect of highly dispersed metallic species in the vicinity of strong acid sites as it was evidenced by XRD and FT-IR spectroscopic investigations. The incidence of both Brønsted and Lewis acid sites has beneficial effect on acidity of the zeolite enhancing the catalytic activity [31,33].

**Figure 10:**
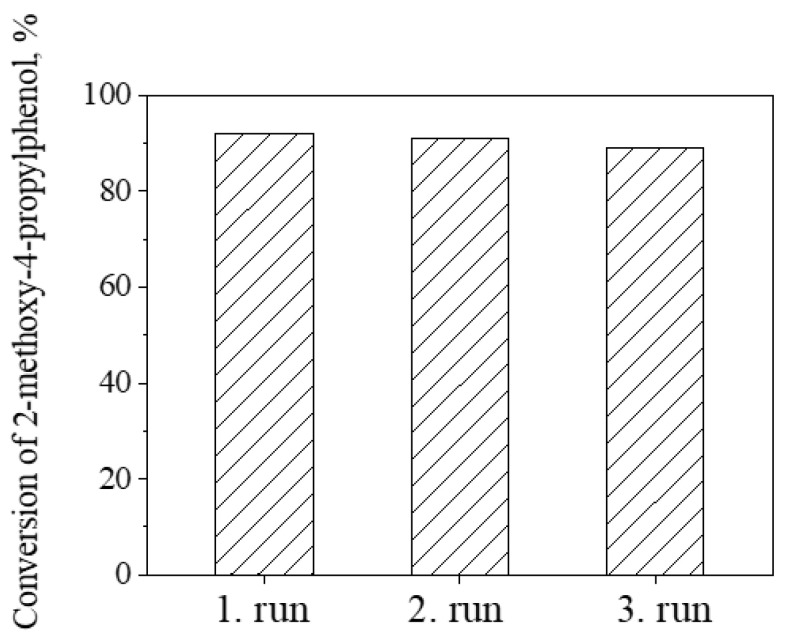
Catalytic activity of 5Ru1Pt-modified Beta zeolites in hydrodemethoxylation and dealkylation of 2-methoxy4-propylphenol at 300 °C in 3 reaction cycles.

The stability of the 5Ru1Pt/HB was tested in three reaction cycles and the data are presented in Figure 10. Catalyst deactivation due to coke formation is negligible. Improved diffusion of reactant and phenol products in beta nanoparticles reduces coke formation.

The XRD data of the spent catalysts do not show any presence of crystalline metal particles (Figure S4). In situ reduction in the metal oxides before the catalytic experiments leads to redistribution of the metal nanoparticles on the zeolite surface, not detectable by XRD.

## 3. Experimental

### 3.1. Materials

The reagents used for the preparation of zeolite Beta were sodium hydroxide (NaOH, Sigma, St. Louis, MO, USA), aluminum isopropylate (Al(O-CH-(CH_3_)_2_)_3_, Sigma), 30 wt. % colloidal silica suspension (Bindzil 30/360 Akzo Nobel), tetraethylammonium hydroxide, 20 wt. % aqueous solution (TEAOH, Sigma). All chemicals were used as received without any purification.

### 3.2. Synthesis of Nanosized Beta Zeolite

Zeolite Beta nanoparticles were synthesized according to Landau et al. [34]. In brief, the synthesis procedure includes the preparation of a precursor gel with the following molar composition: 25.00SiO_2_:0.25Al_2_O_3_:9.0R:0.35Na_2_O:295H_2_O, where R is the organic templating agent tetraethylammonium hydroxide (TEAOH). The crystalline sample was recovered after 9 days of hydrothermal treatment of the gel at 100 °C. The solid phase was washed and centrifuged with deionized water repeatedly until pH 7 was reached then dried by freeze-drying. Organic structure-directing agent was removed by calcination at 550 °C for 6 h in static air to get the H-form of the zeolite. The obtained zeolite contained 0.97 mmol/g_calc_ Al and 0.07 mmol/g_calc_ Na, as determined by ICP-OES method by its microwave digesting in HF/HNO_3_ mixture. The water content was 5.5 wt. %. The bulk Si/Al ratio was 16.3, and the ion-exchange capacity is 0.83 mmol/g_calc_, determined by temperature-programmed evolution of ammonia from the NH_4_ form of zeolite Beta. The sample was denoted as HB.

### 3.3. Impregnation with Ni, Ru, or/and Pt Nanoparticles of Nanosized Beta Zeolite

**Scheme 2:**
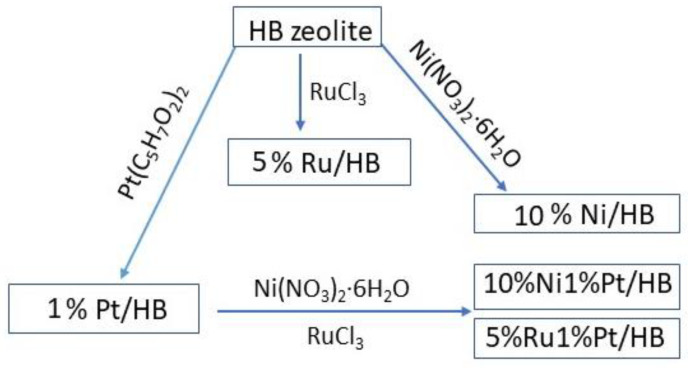
Preparation of the catalysts.

Wet impregnation technique with nickel, ruthenium, and platinum salts was applied for loading of 10 wt. %, 5 wt. %, and 1 wt. % metals, respectively (Scheme 2). The HB support was dehydrated at 160 °C for 2 h before impregnation procedure.

Ni-modified Beta zeolite was prepared by the following procedure: 550 mg Ni(NO_3_)_2_∙6H_2_O was dissolved in 16.5 mL ethanol and was added to 1 g HB support by stirring until evaporation of the solvent. Then the sample was dried at 80 °C for 18 h and calcined at 450 °C for 3 h with a rate of 1°C/min. The sample was denoted as 10Ni/HB.

Pt-modified Beta zeolite was prepared by dissolving of 20 mg Pt(II) acetyl acetonate in 20 mL ethanol and then the solution was added to 1 g HB support by stirring at room temperature for 1 h. The ethanol solvent was removed in vacuum evaporator at 40 °C. Then the sample was dried at 70 °C for 18 h and calcined at 450 °C for 3 h with a rate of 1°C/min. The sample was denoted as 1Pt/HB.

Ru-modified HB sample was prepared by dissolving of 107 mg RuCl_3_ in 80 mL water and then the solution was added to 1 g Beta zeolite by stirring at room temperature for 12 h. The solvent was removed in vacuum evaporator at 40 °C. Then the sample was dried at 110 °C for 12 h and calcined at 450 °C for 3 h with a rate of 1 °C/min. The sample was denoted as 5Ru/HB.

Bimetallic Pt- and Ni-modified Beta zeolite was prepared by the following procedure: 550 mg Ni(NO_3_)_2_∙6H_2_O dissolved in 4 6.6 mL ethanol was added to 1 g 1Pt/HB sample and then was dried at 80 °C for 18 h. The precursor salt was decomposed in air at 450 °C with a rate of 1°C/min for 3 h. The sample was denoted as 10Ni1Pt/HB.

Bimetallic Ru- and Pt-modified HB sample was prepared dissolution of 109 mg RuCl_3_ in 16.5 mL water and was added to 1 g 1Pt/HB sample and dried at 80 °C for 18 h. The precursor salt was decomposed in air at 450 °C with a rate of 1°C/min for 3 h. The sample was denoted as 5Ru1Pt/HB.

### 3.4. Characterization

X-ray powder diffraction patterns were recorded by Philips PW 1810/3710 type (Bruker AXS Advanced X-ray Solutions GmbH, Karlsruhe, Germany) diffractometer applying monochromatized CuK_α_ radiation (40 kV, 35 mA). Patterns were collected between 3 and 75 °2θ with 0.02 ° step size for 1 s. Crystallite size of the metal oxides were determined by the Sherrer equation evaluating the FWMH values of the oxide phases with full profile fitting method. For the identification of crystalline phases ICDD database was used with card No. for zeolite Beta: 00-047-0183 RuO_2_: 43-1027, NiO: 78-643.

Specific surface area and pore volume of the samples was determined from N_2_ physisorption isotherms collected at −196 °C using AUTOSORB iQ-C-MP-AG-AG (Quantachrome Instruments, Anton Paar brand, Boynton Beach, FL, USA). Samples were pre-treated at 350 °C in vacuum before nitrogen adsorption. Total pore volume was determined according to the Gurvich rule at relative pressure of 0.9.

The temperature-programmed reduction-thermogravimetric analysis (TPR-TGA) investigations were performed by a STA449F5 Jupiter type instrument of NETZSCH Gerätebau GmbH (Netzsch, Germany). In a typical measurement 20 mg of sample was placed in a microbalance crucible and heated in a flow of 5 vol. % H_2_ in Ar (100 cm^3^/min) up to 500 °C at a rate of 5 °C/min and a final hold-up of 1 h. Prior to the TPR experiments the samples were treated in situ in at 500 °C in air flow (10 °C/min) for 1 h.

TEM images were taken on JEOL JEM 2100 type transmission electron microscope (JEOL, Tokyo 196-8558, Japan) at 200 kV accelerating voltage. Samples were suspended in a small amount of ethanol and a drop of the suspension was deposited onto a copper grid covered by amorphous carbon supporting film and dried at ambient conditions.

NMR spectra were recorded on a Bruker Avance II+ 600 NMR spectrometer (Bruker, Karlsruhe, Germany) operating at 600.01 MHz ^1^H frequency (119.21 MHz for ^29^Si, 156.34 MHz for ^27^Al), using 4 mm solid-state CP/MAS dual ^1^H/^31^P-^15^N probehead. The samples were packed in 4 mm rotors (Zr_2_O) and spun at magic angle spinning (MAS) rate of 10 kHz for the measurement of ^29^Si spectra and at 14 kHz for the ^27^Al spectra. The quantitative ^29^Si NMR spectra were acquired with single-pulse sequence, 90° pulse length of 4.5 μs, time domain data points of 3 K, spectrum width of 29 kHz, 400 transients were accumulated with a relaxation delay of 120 s between each scan. The spectra were zero filled to 16 K data points and processed with an exponential window function (line broadening factor 10) before the Fourier transformation. ^27^Al spectra were acquired with single-pulse sequence, 90° pulse length of 2.8 μs, 128 K time domain data points, spectrum width of 780 kHz, 1024 scans, and a relaxation delay of 0.5 s. All spectra were processed with an exponential window function (line broadening factor 10). The following experimental parameters were used for measuring the ^1^H→^29^Si cross-polarization MAS (CP MAS) spectra: ^1^H excitation pulse of 3.6 μs, contact time of 2 ms, 5 s relaxation delay, more than 4000 scans, MAS rate 10 kHz. ^1^H SPINAL-64 decoupling scheme was used during acquisition of CP experiments.

FT-IR spectra were measured by a Nicolet Compact 6700 spectrometer (Thermo Fisher Scientific, Waltham, MA, USA). Self-supported wafers (10 × 20 mm) were pressed and contacted with pyridine (Py, 6 mbar) as probe molecule. By placing the pellets into the special IR cell, samples were dehydrated at 400 °C in high vacuum for 1 h. Py was adsorbed on substances for 30 min at 200 °C, followed by subsequent evacuation at 100, 200, 300, and 400 °C for 30 min. Py was desorbed at different temperatures by evacuation, and a spectrum was recorded at the IR beam temperature with 128 scans at a resolution of 2 cm^−1^. The spectra were normalized to 5 mg/cm^2^ weight of the wafers to make the quantitative comparison possible.

### 3.5. Catalytic Activity Measurements

Prior to the catalytic tests samples were pre-treated for 1 h in hydrogen (60 mL/min) at 400 °C. Hydrodemethoxylation of 2-methoxy-4-propylphenol was studied at atmospheric pressure using a fixed-bed flow-through reactor with 30 mL/min hydrogen stream. In the reaction, the 50 mg sample (particle size 0.2–0.8 mm) was tested, and diluted with 50 mg glass beads of the same diameter, previously checked to be inactive. The reactor itself was a quartz tube of 15 mm inner diameter, with the catalyst bed at the middle. A thermocouple was positioned in the catalyst bed for accurate measurement of the catalyst temperature. All gas lines of the apparatus were heated continuously at 110 °C in order to minimize reactant and products adsorption on the tube walls. The gas stream passed through a saturator filled with 2-methoxy-4-propylphenol equilibrated at 80 °C. The reaction steady state was established after 30 min in each temperature. On-line analysis of the reaction products was performed using on NEXIS GC-2030 ATF (Shimadzu Group Company, Kyoto, Japan) with a 30 m HP 5MS capillary column.

## 4. Conclusions

Mono- and bimetallic Ni, Ru, and Pt-containing nanosized Beta zeolite catalysts were successfully prepared by impregnation procedure. Nanosized metal (Ni, Ru, and Pt) particles were detected in the mono- and bimetallic catalysts. The incorporation of nickel ions into the zeolite matrix by the applied impregnation method was found. The obtained catalysts show high activity in hydrodemethoxylation/dealkylation of 2-methoxy-4-ethylphenol to phenol. Among the studied catalysts, 5Ru1Pt/HB shows the highest activity, selectivity, and stability for formation of phenol by hydrodeoxygenation/dealkylation of 2-methoxy4-propylphenol obtained from lignin depolymerization.

## Figures and Tables

**Table 1:** Textural and other physico-chemical properties of the obtained catalysts.

Samples	BET Surface Area, (m^2^/g)	Pore Volume, (cm^3^/g)	Crystallite Size, (nm)	Reducibility ^a^, (%)
Beta	627	0.46	-	-
10Ni/HB	577	0.34	5-NiO	90
10Ni1Pt/HB	584	0.36	26-NiO	100 (100) ^b^
1Pt/HB	604	0.42	˂5	100 ^b^
5Ru/HB	579	0.36	26-RuO_2_	96 ^c^
5Ru1Pt/HB	598	0.40	29-RuO_2_	100 (100) ^b^

^a^ Calculated from TPR curve in the range of 150–600 °C, based on the total weight loss related to the calculated theoretical weight loss for the reduction in the corresponding oxides to metallic state; ^b^ reducibility for PtO_2_; ^c^ calculated from TPR2 curve after the TPR1-TPO-TPR2 experiments in the range of 150–600 °C.

**Table 2:** Area of the signals of the different Si structural units, obtained by deconvolution of the spectral patterns in the single pulse ^29^Si NMR spectra, and the relative fractions of FAl and EFAl species, determined from 27Al NMR spectra.

Sample	Si(1OH) −101 ppm	Si(1Al)		Si(0Al)		Si/Al	FAl, %	EFAl%
		−105 ppm	−107 ppm	−110 ppm	−114 ppm			
Beta	19 *	19	-	49	13	21	80	20
10Ni/HB	9	11	15	59	6	15	95	5
10Ni1Pt/HB	14	17	10	52	7	15	86	14
1Pt/HB	6	12	19	54	9	13	81	19
5Ru/HB	12	13	15	52	9	14	80	20
5Ru1Pt/HB	15	12	10	55	7	18	83	17

* The deconvoluted spectrum of parent zeolite Beta shows two resonances at −100 ppm and −102 ppm assigned for silanol groups with total relative area given in the table.

## Data Availability

Not applicable.

## Supplementary Materials

The following are available online, Figure S1 ART FTIR spectra of the initial and modified zeolites, Figure S2: Bright Field TEM (BFTEM) images of 5Ru/HB at magnification 10000× (**a**) and 40000× (**b**). SAED patterns (**c**), HRTEM images (**e**, **f**) and the corresponding Fast Fourier Transform (FFT) patterns (**d**, **g**) of 5Ru/HB sample, Figure S3. Comparison of the ^1^H→^29^Si CP (black) and single pulse (red) ^29^Si NMR spectra of samples 10Ni/HB and Beta. The lower signal-to-noise (S/N) ratio for the ^1^H→^29^Si CP spectra as compared to the single pulse experiments indicates the low efficiency of the magnetization transfer due to generally low amount of silanol groups present in the samples, Figure S4. XRD of the spent catalysts.

## Sample Availability

Samples of Beta zeolites are available from the authors.
