# Supplementary material for: Hydrodemethoxylation/Dealkylation on Bifunctional Nanosized Zeolite Beta
Source: Molecules. 2021 Dec 20;26(24):7694. doi: 10.3390/molecules26247694 (PMC8708772; doi:10.3390/molecules26247694)
Supplement: Supplementary file 1 [file molecules-26-07694-s001.zip › molecules-1495479-supplementary.pdf]

Supplementary materials

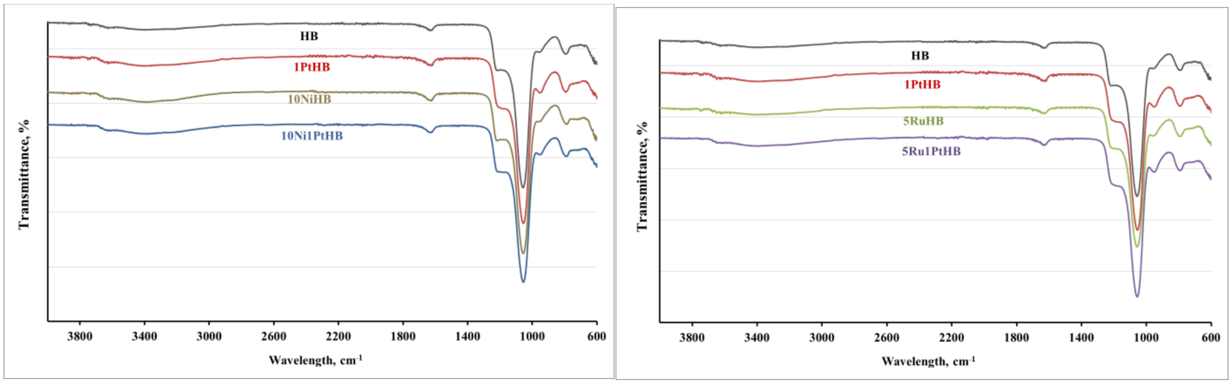

Figure S1. ART FTIR spectra of the initial and modified zeolites.

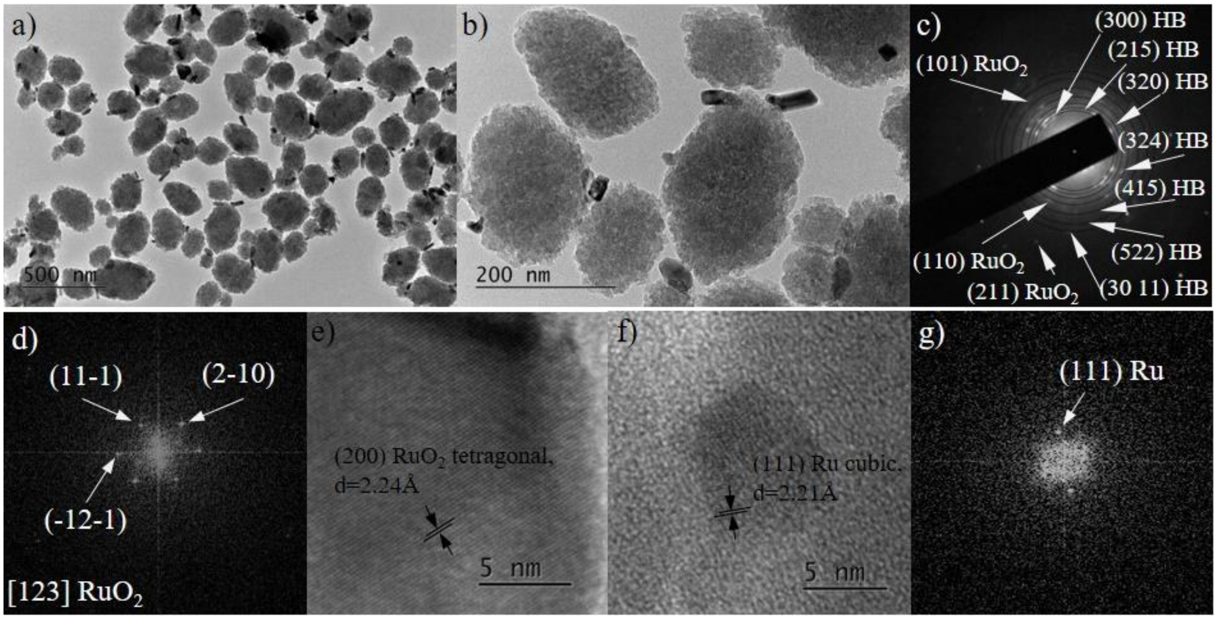

Figure S2. Bright Field TEM (BFTEM) images of 5Ru/HB at magnification 10000x (a) and 40000x (b). SAED patterns (c), HRTEM images (e, f) and the corresponding Fast Fourier Transform (FFT) patterns (d, g) of 5Ru/HB sample.

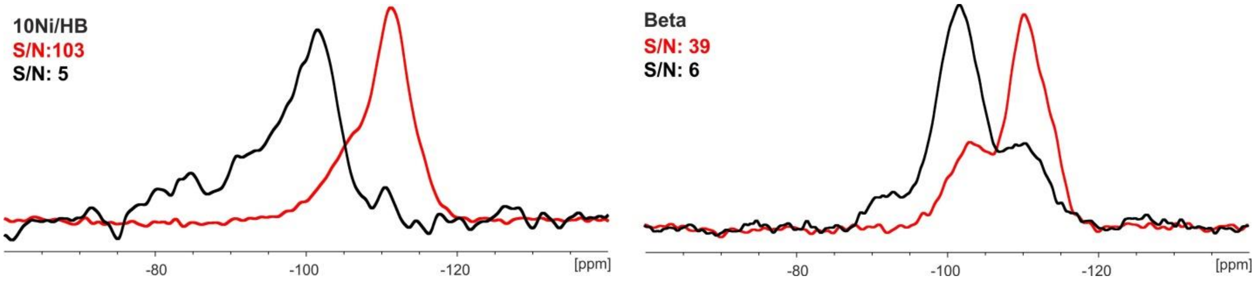

Figure S3. Comparison of the $^{1}H \rightarrow ^{29}Si$ CP (black) and single pulse (red) ^{29}Si NMR spectra of samples 10Ni/HB and Beta. The lower signal-to-noise (S/N) ratio for the $^{1}H \rightarrow ^{29}Si$ CP spectra as compared to the single pulse experiments indicates the low efficiency of the magnetization transfer due to generally low amount of silanol groups present in the samples.

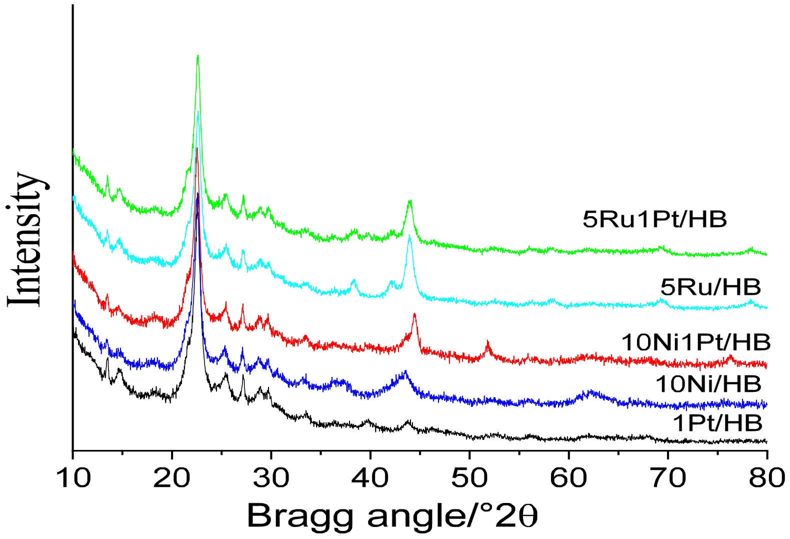

Figure S4. XRD of the spent catalysts.
